# Evaluation of the Soda Tax on Obesity and Diabetes in California: A Cost-Effectiveness Analysis

**DOI:** 10.1177/23814683241309669

**Published:** 2025-01-13

**Authors:** Fan Zhao, Risha Gidwani, May C. Wang, Liwei Chen, Roch A. Nianogo

**Affiliations:** Department of Epidemiology, Fielding School of Public Health, University of California, Los Angeles (UCLA), Los Angeles, California, USA; Department of Health Policy and Management, Fielding School of Public Health, University of California, Los Angeles (UCLA), Los Angeles, California, USA; RAND Corporation, Santa Monica, CA, USA; Department of Community Health Science, Fielding School of Public Health, University of California, Los Angeles (UCLA), Los Angeles, California, USA; Department of Epidemiology, Fielding School of Public Health, University of California, Los Angeles (UCLA), Los Angeles, California, USA; Department of Epidemiology, Fielding School of Public Health, University of California, Los Angeles (UCLA), Los Angeles, California, USA; California Center for Population Research (CCPR), Los Angeles, California, USA

**Keywords:** soda tax, obesity, diabetes, cost effectiveness analysis, microsimulation, California

## Abstract

**Introduction.** Consumption of sugar-sweetened beverages (SSBs) contributes to weight gain, obesity, and diabetes. Soda tax has been proposed to reduce consumption of SSBs. What remains unclear is whether the soda tax has an effect on health and health care costs. We evaluated the cost-effectiveness of a 1-cent-per-ounce soda tax on obesity and diabetes in California. **Methods.** A microsimulation state-transition model was used to evaluate the cost-effectiveness of the soda tax. Health outcomes were measured in quality-adjusted life-years (QALYs). Health care costs were projected from 2015 to 2035. **Results.** In a simulated cohort of Californian adults, the soda tax policy prevented 2.28 million cases of overweight (95% confidence interval [CI] −0.06 to 6.63) and 0.49 million cases of obesity (95% CI −0.19 to 1.18). From the health care perspective, the incremental cost-effectiveness ratio of the soda tax was $124,839 dollars per QALY (95% CI −1,151,983 to 557,660). From the health care perspective, the soda tax policy was cost-effective 80% of the time in the probabilistic sensitivity analysis using a willingness-to-pay threshold of $100,000 per QALY. **Conclusions.** The 1-cent-per-ounce soda tax reduced the number of obesity cases, diabetes cases, and related complications. In addition, the soda tax policy implemented in California was cost-effective most of the time.

**Highlights:**

- Question: What remains unclear is whether the soda tax has an effect on health and health care costs.
- Findings: The 1-cent-per-ounce soda tax reduced the number of obesity cases, diabetes, and related complications. In addition, the soda tax policy brought large amounts of revenue.
- Meaning: This study provides additional evidence regarding the health care costs and cost-effectiveness related to the implementation of a soda tax.

## Introduction

Obesity is a major public health problem in the United States, and its prevalence increased from 30.5% to 41.9% from 2000 to 2020.^1^ Medical costs for adults who have obesity are significantly higher than for adults with a healthy weight.^1^ Obesity-related conditions include heart disease, stroke, diabetes, and certain types of cancer.^1^ About 1 in 10 Americans have diabetes, and approximately 90% to 95% of them are type 2 diabetes.^2^

It has been well established that sugar-sweetened beverage (SSB) consumption increases the risk of weight gain, obesity, diabetes, and cardiovascular diseases (CVDs).^3,4^ SSBs are beverages containing added caloric sweeteners such as brown sugar, corn sweetener, corn syrup, dextrose, fructose, glucose, high-fructose corn syrup, honey, lactose, malt syrup, maltose, molasses, raw sugar, and sucrose.^5^ They include soft drinks, fruit drinks, energy drinks, sweetened waters, and presweetened coffees and teas.^6^ SSBs contribute to more than half of added sugar in the American diet.^7^ On average, American youth consume 143 calories from SSBs, and US adults consume about 145 calories from SSBs on any given day.^5^ The average US household spends an estimated $850 annually on soft drinks, for a total of $65 billion alone in 2010.^8^

One proposed solution to reducing SSB consumption is a soda excise tax, which is implemented as a fee per beverage unit (e.g., 1-cent-per-ounce) and imposed on the manufacturers, in contrast to sales taxes that are collected from the consumer at purchase.^9–13^ Berkeley, California, was the first US jurisdiction to pass a 1-cent-per-ounce soda tax in 2014.^14^ Albany, Oakland, and San Francisco in California approved a similar 1-cent-per-ounce tax in 2016.^15,16^ Evidence in these cities showed that the soda tax reduces SSB purchases as well as intake.^17,18^

However, it remains unclear whether the soda tax has an effect on health and health care costs.^19^ There has been debate on whether the soda tax should be implemented at the state or federal level from beverage companies. Several states have passed legislation that preempts new local soda taxes.^20^ California’s preemption law, which went into effect on June 28, 2018, banned more local soda taxes.^20^

The objective of this study is to assess the cost-effectiveness of a 1-cent-per-ounce soda excise tax on preventing obesity and diabetes in California, where health outcomes were evaluated in 2 ways: 1) cases of health conditions prevented and 2) quality-adjusted life-years (QALYs). The expected total revenue from the tax was also calculated to inform the ongoing policy debate.^21^

## Methods

We implemented a microsimulation state-transition model to evaluate the cost-effectiveness of the soda tax in California. We followed the best practices outlined in the Second Panel on Cost-Effectiveness in Health and Medicine.^22^ We followed the consolidated Health Economic Evaluation Reporting Standards (CHEERS) statement for the reporting of health economic evaluations^23^ (Supplement Table 1). The model had 2 arms: a soda tax arm and a “do nothing” arm. The model took a health care system and government perspective.

### Target Population and Setting

Our target population was Californian adults who were 18 y or older in 2015, the first year in which the soda tax took effect.^24^ We used data from the California Health Interview Survey (CHIS), which is an annual cross-sectional survey of California households.^1^ The CHIS sample is weighted to be representative of the noninstitutionalized population living in households in California.^25^ A detailed description of the sampling and weighting process is available in the original report of the survey.^25^ Body mass index (BMI; kg/m^2^) was calculated from self-reported height and weight and used to assess overweight (25 ≤ BMI < 30) and obesity (30 ≤ BMI) status.^1^

There were 29,247,121 adults in California in 2015.^26^ There were 20,511 adults surveyed in CHIS 2015. We ran a model based on 20,000 individual hypothetical people and rescaled the results to the actual number of adults in California in 2015 by multiplying the model estimates by 1,462.36, which was equal to 29,247,121 divided by 20,000. To assign baseline weight and height to simulated individuals, we randomly sampled from the joint probability distributions of weight and height to preserve the correlation and heterogeneity between individuals at baseline.

### Soda Tax Arm and Base Case

**Figure 1:**
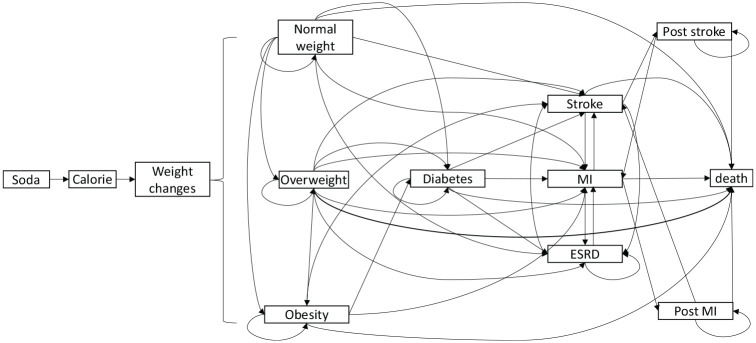
Markov model for cost-effectiveness analysis of a one-cent-per-ounce soda tax. The soda tax affected soda consumption, which affected added sugar intake, calorie intake, and weight change. MI, myocardial infarction; ESRD, end stage renal diseases. The cycle length is 1 year and the time horizon of the model is 20 years.

We assumed the soda tax was applied in retail business entities including restaurants and vending machines. We evaluated the impact of the soda tax (compared with no soda tax) in the development of overweight, obesity, diabetes, related complications, and death (Figure 1).

To compute the weight change due to soda tax, we applied the estimate from a longitudinal household survey of beverage consumption in Oakland 1 y after the implementation of the 1-cent-per-ounce soda tax.^27^ In the study, beverage consumption was estimated using the National Health and Nutrition Examination Survey (NHANES) Dietary Screener Questionnaire, then the total amount of added sugar from beverages was constructed from the National Cancer Institute algorithm.^27^ Daily grams of added sugar from SSBs reduced 3.08 g (95% confidence interval [CI], −10.27, 4.11) among adults. There are roughly 4 calories in 1 g of sugar. Therefore, the amount of sugar converted to roughly 12.32 fewer calories per day (95% CI −41.08, 16.44). We estimated the weight loss with a static calorie-to-weight model in which every pound of body weight loss requires an energy deficit of about 3,500 calories.^28^ Thus, a 12-calorie reduction in consumption was expected to result in a weight change of −0.56 kg (95% CI −1.93, 0.77) each year, which is a weight reduction of 0.56 kg. We assumed that soda consumption and weight would change in the first year of policy implementation. We also assumed that the impact of the soda tax remained consistent over the years.

In the meantime, adults in both arms naturally gained 0.45 to 0.91 kg each year^29^ (Table 1). In the base case, there were no soda taxes.

**Table 1:** Model Input: Weight Loss, Tax Revenue, Cost, Utilities, and Transition Probabilities

Parameter	Value	Source
Natural weight gain, kg/y	0.45 to 0.91	Hutfless, 2013^29^
Weight change due to reduction of soda consumption, kg/y	−0.56 (95% CI −1.93 to 0.77)	Cawley, 2020^27^
Tax revenue from taxed beverages per year	$862,722,888 (±25% 647,042,166 to 1,078,403,610)	Cerrigione, 2022^30^
Soda tax government administration cost per year	$8,627,229 (±25% 6,470,422 to 10,784,036)	Cerrigione, 2022^30^
Health cost per person per year		
Normal weight	0	assumption
Overweight	$1,320 (95% CI 1,069 to 1,607)	Ward, 2021^31^
Obesity	$3,955 (95% CI 3,519 to 4,363)	Ward, 2021^31^
Diabetes	$21,218 (±25% 15,913 to 26,522)	American Diabetes Association, 2018^32^
Stroke	$103,425 (±25% 77,569 to 129,281)	Ward, 2014^33^
Poststroke	$38,159 (±25% 28,619 to 47,698)	Ward, 2014^33^
MI	$138,603 (±25% 103,952 to 173,253)	Ward, 2014^33^
Post-MI	$4,675 (±25% 3,506 to 5,843)	Ward, 2014^33^
ESRD	$176,647 (±25% 132,485 to 220,808)	National Institute of Diabetes and Digestive and Kidney Diseases, 2019^34^
Death	0	
Utilities		
Normal weight	1 (±25% 0.75 to 1)	Assumption
Overweight	0.876 (±25% 0.657 to 1)	Jia, 2009^35^
Obesity	0.850 (±25% 0.637 to 1)	Jia, 2009^35^
Diabetes	0.785 (95% CI 0.681 to 0.889)	Beaudet, 2014^36^
Stroke, poststroke	0.621 (95% CI 0.459 to 0.784)	Beaudet, 2014^36^
MI, post-MI	0.730 (95% CI 0.614 to 0.847)	Beaudet, 2014^36^
ESRD	0.61 (95% CI 0.51 to 0.71)	McManus, 2021^37^
Death	0	
Transition probabilities		
Normal weight → diabetes	0.0069 (95% CI 0.0058 to 0.0083)	American Heart Association, 2022^38^
Normal weight → stroke	0.0028 (95% CI 0.0025 to 0.0032)	American Heart Association, 2022^38^
Normal weight → MI	0.0028 (95% CI 0.0025 to 0.0033)	American Heart Association, 2022^38^
Normal weight → ESRD	0.0004 (±25% 0.0003 to 0.0005)	American Heart Association, 2022^38^
Normal weight → death	0.0090 (±25% 0.0067 to 0.0112)	Centers for Disease Control and Prevention, 2019^39^
Overweight → diabetes	0.0088 (95% CI 0.0084 to 0.0092)	Ganz, 2014^40^
Overweight → stroke	0.0029 (95% CI 0.0027 to 0.0033)	Mitchell, 2015^41^
Overweight → MI	0.0033 (95% CI 0.0028 to 0.0039)	Thomsen, 2014^42^
Overweight → ESRD	0.0005 (95% CI 0.0005 to 0.0006)	Wang, 2008^43^
Overweight → death	0.0087 (95% CI 0.0087 to 0.0087)	Angelantonio, 2016^44^
Obesity → diabetes	0.0135 (95% CI 0.0119 to 0.0154)	Cameron, 2021^45^
Obesity → stroke	0.0035 (95% CI 0.0032 to 0.0039)	Mitchell, 2015^41^
Obesity → MI	0.0044 (95% CI 0.0034 to 0.0055)	Thomsen, 2014^42^
Obesity → ESRD	0.0007 (95% CI 0.0006 to 0.0008)	Wang, 2008^43^
Obesity → death	0.0118 (95% CI 0.0117 to 0.0119)	Angelantonio, 2016^44^
Diabetes → stroke	0.0042 (95% CI 0.0032 to 0.0057)	Gregg, 2014^46^
Diabetes → MI	0.0051 (95% CI 0.0038 to 0.0067)	Gregg, 2014^46^
Diabetes → ESRD	0.0023 (95% CI 0.0022 to 0.0024)	Gregg, 2014^46^
Diabetes → death	0.0128 (95% CI 0.0120 to 0.0137)	Li, 2019^47^
Stroke → MI	0.03 (95% CI 0.01 to 0.05)	Gunnoo, 2015^48^
Stroke → death	0.17 (±25% 0.13 to 0.21)	American Heart Association, 2022^38^
MI → stroke	0.081 (±25% 0.061 to 0.101)	Loh, 1997^49^
MI → death	0.22 (±25% 0.16 to 0.27)	American Heart Association, 2022^38^
ESRD → stroke	0.0172 (95% CI 0.0146 to 0.0200)	Seliger, 2003^50^
ESRD → MI	0.0040 (95% CI 0.0032 to 0.0050)	Beddhu, 2002^51^
ESRD → death	0.034 (±25% 0.025 to0.042)	Centers for Disease Control and Prevention, 2019^39^
Poststroke → MI	0.0167 (95% CI 0.0136 to 0.0198)	Boulanger, 2018^52^
Poststroke → death	0.0173 (95% CI 0.0119 to 0.0235)	Engstad, 2003^53^
Post-MI → stroke	0.0087 (95% CI 0.0083 to 0.0092)	Sundboll, 2016^54^
Post-MI → death	0.21 (±25% 0.16 to 0.26)	Law, 2002^55^

±25%, plus and minus 25% of the value; CI, confidence interval; ESRD, end-stage renal disease; MI, myocardial infarction. → indicates the transition from one heath state to another health state. All costs were calculated in 2022 dollars.

### Microsimulation State-Transition Model

Our microsimulation Markov model simulated the individual trajectories, conditional on previous history of disease.^56,57^ The model had a 1-y cycle length and 20-y time horizon (2015–2035).

Health states included normal weight, overweight, obesity, diabetes, and death. We also included stroke, poststroke, myocardial infarction (MI), post-MI, and end-stage renal disease (ESRD) as diabetes complications. We differentiated first-time stroke from recurrent stroke and first-time MI from recurrent MI because first-time occurrence is more costly.^33^

We followed the Grading of Recommendations Assessment, Development, and Evaluation (GRADE) framework from the Cochrane Collaboration to develop summaries of model input parameters.^58,59^ A summary of inputs and data sources of key model parameters is provided in Table 1. A detailed description of the search strategy can be found in Supplementary Text 1.

### Transition Probabilities

Transition probabilities were obtained from previously published meta-analyses or prospective cohort studies. Following conversion formula denoted in Gidwani and Russell^60^ and VanderWeele,^61^ we derived 1-y transition probabilities from sex-/age-/race-adjusted risks, risk ratios, incidence rate ratios, hazard ratios, or odds ratios (Supplementary Text 2, Supplementary Figure 1).

### Cost and Utility Data

#### Cost data

Soda tax implementation costs included the government administration cost. MUNI Services, a private firm contracted by the city of Berkeley to collect soda tax revenue, charges 2% of the tax proceeds.^21^ Therefore, we estimated the overall cost of implementing the statewide soda tax policy as 2% of the annual soda tax revenue, with 1% for government administration cost.^50^ Annual state revenue was calculated using the UConn Rudd Center for Food Policy and Health Sugary Drink Tax Calculator. This calculator multiplies the average per-person intake of SSB after soda tax implementation by the tax rate and the total adult population.^30^ Beverage sales were assumed to represent beverage consumption. Proprietary data from the Beverage Marketing Corporation was used to measure the total sales of SSB sold across all retail channels in the United States, including all types of food stores, fountain drinks in restaurants, vending machines, and so forth.^30^

Direct medical costs for overweight and obesity included medical expenditures for hospital stays, emergency room (ER) visits, outpatient department visits, office-based medical provider visits, home health care, other medical expenses, and prescription medicines.^31,62^ Direct medical costs for diabetes included institutional care, outpatient care, oral medications, noninsulin injectable antidiabetes agents, hearing devices, eyewear, and prostheses.^32^ Direct medical costs for stroke, MI, and ESRD included inpatient stays, ER visits, outpatient visits, tests and procedures, as well as medications.^33^ Direct medical costs were estimated from the United States Renal Data System 2022 Annual Data Report and the American Diabetes Association 2017 Report^32–34^ (Table 1).

#### Utility data

We used EQ-5D utility to value health.^63^ Normal weight was assigned a utility value of 1 and death a utility value of 0.^51^ The EQ-5D scores for other conditions were 0.876 for overweight, 0.850 for obesity, 0.785 (95% CI 0.681, 0.889) for diabetes, 0.621 (95% CI 0.459, 0.784) for stroke as well as poststroke, 0.730 (95% CI 0.614, 0.847) for MI as well as post-MI, and 0.61 (95% CI 0.51, 0.71) for ESRD.^35–37^

All costs were adjusted for inflation to 2022 dollars using the Personal Consumption Expenditure Price Index.^57^ Both costs and health outcomes were discounted at 3% per year.

### Study Perspectives

Analyses were conducted from the health care sector as well as government perspective, similar to Lee et al.,^22,64^ also in accordance with recommendations from the Second Panel on Cost-Effectiveness in Health and Medicine. For the health care sector perspective, we included only direct medical costs, and for the government perspective, we incorporated government tax collection costs, soda tax revenue, and health care costs.

### Outcomes

Health gains included the number of new diseases prevented, deaths averted, and QALYs gained and cost-effectiveness was assessed using the incremental cost-effectiveness ratio (ICER). The ICER was calculated by dividing the difference in net costs by the difference in net effectiveness. The net costs and net effectiveness were computed comparing the costs and effectiveness (QALYs) in the soda tax scenario with the status quo (no soda tax).^12^ We used a willingness-to-pay (WTP) threshold of $100,000 per QALY and presented the results graphically using cost-effectiveness acceptability curves.^65^

### Sensitivity Analysis

To evaluate the uncertainty around estimates and generate standard errors, we used probabilistic sensitivity analyses, using Monte Carlo sampling with replacement for all input parameters for 1,000 iterations (Table 1). We specified gamma distribution for cost inputs, log-normal distribution for relative risk, and beta distribution for probability and utility.^66,67^ Results were presented as means and 95% CIs.

### Internal and External Validation

We evaluated the internal validity of the model by comparing the weight distribution of the simulated cohort with the CHIS cohort in 2015. We also compared the weight distribution as well as the distribution of clinical outcomes of the simulated cohort with the CHIS cohort in 2019.

We evaluated the external validity of the model by changing the weight and height input to be the NHANES in 2015 and then compared the weight distribution as well as the distribution of clinical outcomes of the simulated cohort with the NHANES cohort in 2019.^68,69^

Data analysis was implemented in R software, version 4.0.2.

### Role of the Funding Source

The funder had no role in study design, data collection or analysis, decision to publish, or preparation of the article.

## Results

### Model Evaluation

The simulated distribution of weight broadly matched the observed distribution of weight from CHIS in 2015. Among 20,511 adults surveyed by CHIS in 2015, the mean weight was 77.67 kg (*s* = 19.96) and 5,794 (28.2%) were obese. In the simulated cohort in 2015, the mean weight was 77.97 kg (18.92) and 7,217 (25.9%) were obese (Supplementary Table 2). The simulation model-generated values also broadly matched the observed distribution of weight and clinical outcomes from the CHIS 2019 and NHANES 2019 database (Supplementary Tables 3 and 4).

### Main Scenario

There would be 1.27 million (95% CI 1.04 to 1.54) new cases of diabetes when there was a soda tax, compared with 1.64 million (95% CI 1.16 to 2.43) new cases of diabetes when there was no soda tax. In addition, 3.28 million (95% CI −0.06 to 6.63) cases of overweight, 0.49 million cases of obesity (95% CI −0.19 to 1.18), 0.19 million new cases of stroke (95% CI −0.14 to 0.52), 0.16 million new cases of MI (95% CI −0.17 to 0.48), and 0.71 cases of deaths (95% CI −0.41 to 1.83) would be prevented from soda tax (Table 2). The total QALYs were 0.39 billion (95% CI 0.39 to 0.40).

**Table 2:** Summary of Comparativeness under Status Quo and Soda Tax for a Hypothetical Cohort of Californian Adults in 20 y (in Millions)^a^

Items	1-Cent-per Ounce Soda Tax	Status Quo	Number of Cases Prevented
Number of overweight cases	10.53 (9.30, 14.08)	13.81 (12.92, 14.35)	3.28 (−0.06, 6.63)
Number of obesity cases	10.61 (10.53, 11.18)	11.10 (10.71, 11.71)	0.49 (−0.19, 1.18)
Number of diabetes cases	1.27 (1.04, 1.54)	1.64 (1.16, 2.43)	0.38 (−0.33, 1.08)
Number of stroke cases	0.45 (0.36, 0.54)	0.64 (0.42, 1.02)	0.19 (−0.14, 0.52)
Number of MI cases	0.83 (0.69, 0.99)	0.99 (0.75, 1.31)	0.16 (−0.17, 0.48)
Number of ESRD cases	0.30 (0.26, 0.34)	0.29 (0.25, 0.33)	−0.01 (−0.07, 0.05)
Number of death cases	2.03 (1.83, 2.23)	2.74 (1.97, 4.00)	0.71 (−0.41, 1.83)

a Mean and 95% confidence interval. ESRD, end-stage renal disease; MI, myocardial infarction.

From a health care perspective, over 20 y, the health care cost was $529.70 billion (95% CI 420.28 to 681.19) in California when there was a soda tax. Health costs when there was no soda tax were $677.09 billion (95% CI 603.52 to 749.83). The total QALYs were 0.39 billion (95% CI 0.39 to 0.39). The resulting ICER was −$124,839 (95% CI −1,151,983 to 557,660) dollars per QALY (Table 3).

**Table 3:** Summary of Cost-Effectiveness under Status Quo and Soda Tax for a Hypothetical Cohort of Californian Adults in 20 y (Cost and QALYs in Billions)

Items	Status Quo	1-Cent-per Ounce Soda Tax	Incremental Changes
Total QALYs	0.39 (0.39, 0.39)	0.39 (0.39, 0.40)	—
Total health care costs	$677.09 (603.52, 749.83)	$529.70 (420.28, 681.19)	—
Health care cost savings per person	—	—	$ 0.01
Policy implementation cost	—	$ 0.17 (0.13, 0.22)	—
Total tax revenue	—	$18,609.77 (18,719.19, 18,458.28)	—
Net costs in health care perspective	—	—	−$147.39
Net costs in government perspective	—	—	−$19,286.69
ICER, health care perspective	—	—	−124,839 (−1,151,983 to 557,660)

ICER, incremental cost-effectiveness ratio; QALY, quality-adjusted life-year.

Data are presented as mean and 95% confidence interval. Negative costs represent net revenues. Costs are shown in 2022 dollars.

The tax revenue from SSBs was $862.72 million per year. Over 20 y, the net revenue for government was $18,609.60 billion (95% CI 18,719.02 to 18,458.11). Soda tax was cost saving for the government, and the ICER was −14,668,299 (95% CI −112,205,426 to −5,7206,440) dollars per QALY (Table 3).

**Figure 2:**
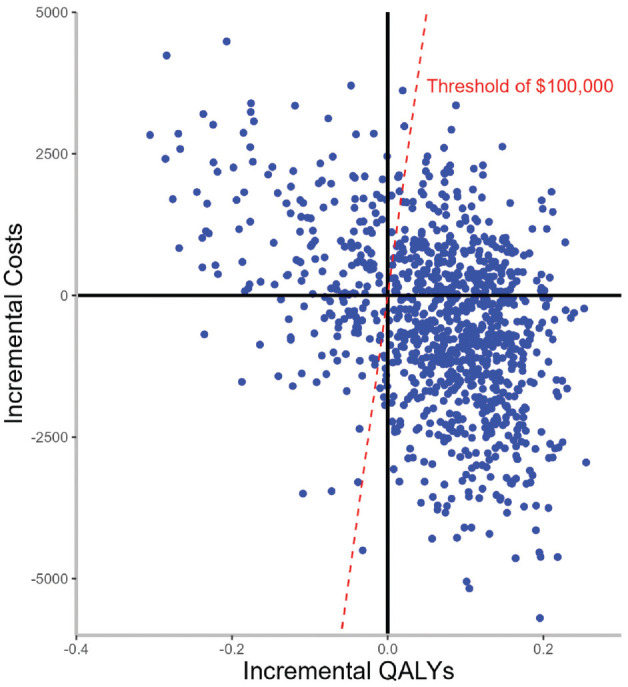
Incremental cost-effectiveness (CE) plane for the 1-cent-per-ounce soda tax on obesity and overweight prevalence. The willingness-to-pay (WTP) threshold was defined as $100,000 per quality-adjusted life-year (QALY), health care perspective.

**Figure 3:**
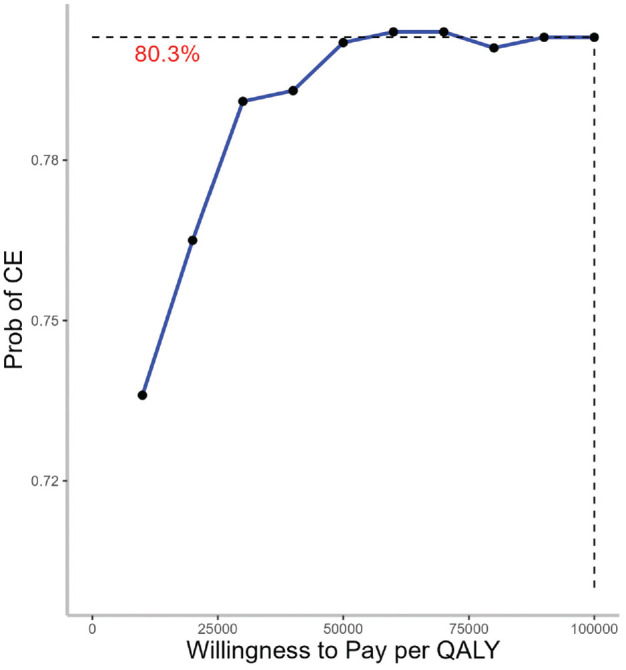
Cost-effectiveness acceptability curve (CEAC) for a 1-cent-per-ounce soda tax, health care perspective. Willingness to pay (WTP) is expressed in 2022 US dollars per quality-adjusted life-year gained ($/QALY). CEAC represents the probability for the 1-cent-per-ounce soda tax to be cost-effective (CE) option for different WTP thresholds.

Based on a WTP of $100,000/QALY, the soda tax policy was cost-effective 80% of the time (Figures 2 and 3).

## Discussion

In this large simulation model of adults in California, we found that a soda tax reduced the number of obesity cases, diabetes, and related CVDs. Second, using the currently known intervention effect, we also found that the soda tax policy implemented in California was cost-effective in reducing the proportion of SSB-related overweight or obesity about 80% of the time from the health care perspective. Lastly, we found that the soda tax policy brought to the government large amounts of revenue totaling $18,609.77 billion over 20 y.

Both feasibility and effectiveness in reducing soda intake are supported by the 7 US cities and locales that have adopted soda tax. Seattle enacted a 1.75-cent-per-ounce soda tax in June 2017. The volume sold of taxed beverages fell in the first year following the tax in Seattle.^70^ Results from Philadelphia are also promising, where the odds of daily consumption of regular soda were lower within the first 2 mo of tax implementation.^71^

Our study adds to the evidence base that instituting a soda tax has the potential of having a significant positive population impact on health and well-being by reducing cases of obesity, diabetes, and consequent complications.^13,21,64,72,73^ Ten-year-long nationwide simulations showed that a 1-cent-per-ounce soda tax reduced health care costs due to obesity.^74^ The 1-cent-per-ounce soda tax was also expected to prevent a significant number of diabetes cases over the lifetime.^64^ Such health benefits seemed to be greater when the amount of soda tax increased. A 2-cent-per-ounce soda tax was expected to prevent more obesity cases than 1-cent-per-ounce soda tax.^75,76^ Therefore, it was possible to raise the amount of soda tax to further influence SSB consumption, thereby reducing calorie intake and weight.

Compared with other policies (e.g., the elimination of soda from vending machines or having health-related warning labels on soda packaging), the soda tax has the advantage of also generating significant revenues.^77^ In our study, the proposed 1-cent-per-ounce soda tax was estimated to generate $862.72 million per year in California alone. Estimates from other data sources showed a similar large amount of revenues.^13,78^ These revenues could be reinvested in preventive health activities. For instance, in Berkeley, California, revenue from soda tax has been allocated to school and community programs to promote healthy eating, increasing public support for such taxes.^24^ Such reinvestment in prevention activities has the potential to produce larger health benefits. This revenue characteristic makes it a highly desirable option for policy makers weighing the costs and benefits of alternative approaches for addressing the urgent public health issue of obesity.

Basu et al.^79^ and Long et al.^13^ illustrated that a soda tax is more cost-effective than soda bans and restrictions. Studies have also shown that a soda tax resulted in more health benefits and saved more on health care costs compared with a fruit and vegetable subsidy, reduced tax subsidy on TV advertising, and active physical education policies.^76,80^ Societal cost savings were greater than $100,000 per 10,000 residents over 10 y when other health conditions—specifically coronary heart disease, cerebrovascular accident, chronic kidney disease, and dental caries and periodontal disease—were also included.^72^

### Strengths and Limitations

Our study has several strengths. We used state-representative data inputs. We assessed 20-y health effects, costs, and cost-effectiveness from health care as well as government perspectives. We modeled the impact of uncertainty in probabilistic sensitivity analyses.

Our project has some limitations. First, in the current study, we did not assess the soda tax’s cost-effectiveness from a societal perspective because soda tax revenues, which are of interest here, are costs that transferred from the public to the government and are not recommended to be included in the societal perspective.^74^ Viable indirect cost estimates for conditions including MI, stroke, obesity, and overweight in the current literature were also not available. Second, there is strong evidence that changes in soda intake were also directly related to diabetes, stroke, and MI independent of weight change. Other mechanisms, such as hyperglycemia, dyslipidemia, inflammation, or endothelial dysfunction, underlie the association.^81–83^ However, we focused on the effects of soda consumption on health outcomes through changes in weight. Therefore, our findings could represent an underestimation of health benefits and cost savings. Third, our results were based on a simulation model that incorporated a broad range of data inputs. While we included the best available evidence on population characteristics, trajectories of obesity prevalence, and obesity-related health care costs, our ability to forecast the precise impacts of soda tax was limited by the uncertainty around each of these inputs and by the assumptions required to build the model. We conducted probabilistic sensitivity analysis to evaluate the extent to which model input uncertainties affect the results. Fourth, we assumed that the effect of soda tax was the same across baseline characteristics (e.g., weight and gender). This was unrealistic because people from different backgrounds could be affected differently and, for example, adjust soda consumption based on their health status. Respondents in the study where we derived estimate of reductions of SSB consumption following soda tax were mostly middle-aged females, who consumed fewer SSB than younger populations did.^27,84^ Therefore, our results could represent an underestimation of the impact of a 1-cent-per-ounce soda tax on weight.

## Conclusion

The 1-cent-per-ounce soda tax reduced the number of obesity cases, diabetes, and related CVDs. In addition, the soda tax policy implemented in California was cost-effective most of the time from the health care perspective. This study provides additional evidence regarding the health care costs and cost-effectiveness related to the implementation of a soda tax.

## Supplemental Material

sj-docx-1-mpp-10.1177_23814683241309669 – Supplemental material for Evaluation of the Soda Tax on Obesity and Diabetes in California: A Cost-Effectiveness Analysis Supplemental material, sj-docx-1-mpp-10.1177_23814683241309669 for Evaluation of the Soda Tax on Obesity and Diabetes in California: A Cost-Effectiveness Analysis by Fan Zhao, Risha Gidwani, May C. Wang, Liwei Chen and Roch A. Nianogo in MDM Policy & Practice
