# Supplementary material for: Evaluation of the Soda Tax on Obesity and Diabetes in California: A Cost-Effectiveness Analysis
Source: MDM Policy Pract. 2025 Jan 13;10(1):23814683241309669. doi: 10.1177/23814683241309669 (PMC11726502; doi:10.1177/23814683241309669)
Supplement: sj-docx-1-mpp-10.1177_23814683241309669 – Supplemental material for Evaluation of the Soda Tax on Obesity and Diabetes in California: A Cost-Effectiveness Analysis [file sj-docx-1-mpp-10.1177_23814683241309669.docx]

**APPENDIX**

**Evaluation of the Soda Tax on Obesity and Diabetes in California: A Cost-effectiveness Analysis**

**Contents**

[**Supplement Text 1** Model input searching strategies. 2](#_Toc172914690)

[**Supplement Text 2** Calculation of transition probability. 2](#_Toc172914691)

[**Supplement Figure 1** Steps to calculate transition probability. 3](#_Toc172914692)

[**Supplement Table 1**CHEERS checklist. 3](#_Toc172914693)

[**Supplement Table 2** Comparison of weight distribution of CHIS and simulated cohort in 2015.* 7](#_Toc172914694)

[**Supplement Table 3** Comparison of weight distribution and clinical outcomes of CHIS and the simulated cohort in 2019, % and 95%CI.* 7](#_Toc172914695)

[**Supplement Table 4** Comparison of weight distribution and clinical outcomes of NHANES and the simulated NHANES cohort in 2019, % and 95%CI. 8](#_Toc172914696)

[**Supplement Table 5** Summary of comparativeness under status quo and soda tax for a hypothetical cohort of 20,000 Californian adults in 20 years.* 8](#_Toc172914697)

[**Supplement Table 6** Summary of cost effectiveness under status quo and soda tax for a hypothetical cohort of 20,000 Californian adults in 20 years.* 9](#_Toc172914698)

[**References** 10](#_Toc172914699)

**Supplement Text 1** Model input searching strategies.

To identify parameter estimates for the simulation model, we conducted a comprehensive literature search in PubMed. To be included, studies had to be quantitative, written in English, published in peer reviewed journals.

For transition probabilities, we first prioritized US-based papers that were evaluating health risks among normal weight, overweight, obese, diabetes without complications, stroke, post stroke, myocardial infarction (MI), post MI, end-stage renal disease (ESRD), and death. When we were unable to find papers in the US, we expanded the search to include studies based in other high-income countries. We ended up including two studies from Denmark and Norway.^1,2^

For cost and utilities, we limited it to US-based papers only.

Studies were also prioritized based on the following study designs (1) latest health report from health organizations or government, (2) latest large cohort studies, (3) meta analyses, (4) cross sectional studies, (5) model simulations.

**Supplement Text 2** Calculation of transition probability.

When risk ratio (RR) is available, $RR=\frac{P_{1}}{P_{0}},$ where $P_{1}$is the probability of the event in exposed group, $P_{0}$is the probability of the event in unexposed group. Therefore, $P_{1}=RR*P_{0}.$ We assume $P_{0}$is the probability in the normal weight group. For example, when we have $RR\left( MI | Overweight \right)=1.260,$ we assume $RR\left( MI | Overweight \right)=\frac{P(MI|overweight)}{P(MI|normal weight)},$ therefore, $P\left( MI | overweight \right)=RR\left( MI | Overweight \right)* P\left( MI | normal weight \right).$

When odds ratio (OR) is available, $odds=\frac{P}{1-P}, OR=\frac{{odds}_{1}}{{odds}_{0}}=\frac{P_{1}/(1-P_{1})}{P_{0}/(1-P_{0})}, RR=\frac{OR}{1-P_{0}+P_{0}*OR}$,.^5^

When hazard ratio (HR) is available, we approximated RR by $\frac{1-{0.5}^{sqrt(HR)}}{1-{0.5}^{sqrt(1/HR)}}$.^6,7^

Converting Probabilities to the cycle length of one year.^5^ $r=\frac{-In(1-P_{1})}{t_{1}}, P_{new}=1-exp\left( -r*t_{2} \right),$where r is rate, $t_{1}$is time frame available, $t_{2}$is time frame we want, here it’s 1 year, and P is probability.

For probabilistic sensitivity analysis, we specified a gamma distribution for cost inputs, log-normal distributions for IRR, RR, HR and OR, and beta distributions for probabilities and utilities.^59^

**
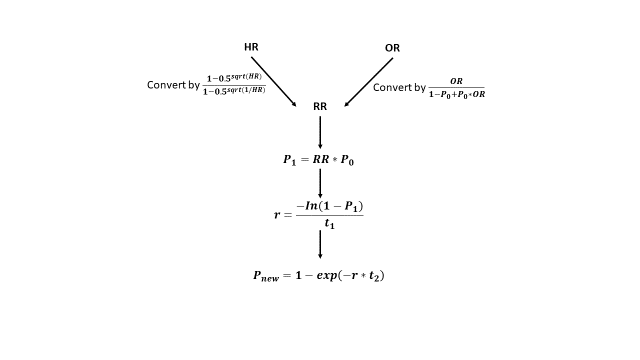
**

**Supplement Figure 1** Steps to calculate transition probability.

**Supplement Table 1**CHEERS checklist.

| **Section/item** | **Item No** | **Recommendation** | **Report**  **Location** |
| --- | --- | --- | --- |
| **Title and abstract** | | | |
| Title | 1 | Identify the study as an economic evaluation or use more specific terms such as “cost-effectiveness analysis”, and describe the interventions compared. | Title. |
| Abstract | 2 | Provide a structured summary of objectives, perspective, setting, methods (including study design and inputs), results (including base case and uncertainty analyses), and conclusions. | Abstract. |
| **Introduction** | | | |
| Background and objectives | 3 | Provide an explicit statement of the broader context for the study. | Introduction paragraphs 1-4. |
|  |  | Present the study question and its relevance for health policy or practice decisions. | Introduction paragraph 5. |
| **Methods** | | | |
| Target population and subgroups | 4 | Describe characteristics of the base case population and subgroups analysed, including why they were chosen. | Method section ‘Target population and setting’; Result section ‘Model validation’. |
| Setting and location | 5 | State relevant aspects of the system(s) in which the decision(s) need(s) to be made. | Introduction paragraph 4. |
| Study perspective | 6 | Describe the perspective of the study and relate this to the costs being evaluated. | Method section ‘Study perspective and cost data’. |
| Comparators | 7 | Describe the interventions or strategies being compared and state why they were chosen. | Method section ‘Comparators’. |
| Time horizon | 8 | State the time horizon(s) over which costs and consequences are being evaluated and say why appropriate. | Method section ‘Microsimulation state-transition model’. |
| Discount rate | 9 | Report the choice of discount rate(s) used for costs and outcomes and say why appropriate. | Method section ‘Utility data’. |
| Choice of health outcomes | 10 | Describe what outcomes were used as the measure(s) of benefit in the evaluation and their relevance for the type of analysis performed. | Method section ‘Microsimulation state-transition model’. |
| Measurement of effectiveness | 11a | *Single study-based estimates:* Describe fully the design features of the single effectiveness study and why the single study was a sufficient source of clinical effectiveness data. | Not applicable. |
|  | 11b | *Synthesis-based estimates:* Describe fully the methods used for identification of included studies and synthesis of clinical effectiveness data. | Supplement section ‘Supplement Text 1’. |
| Measurement and valuation of preference based outcomes | 12 | If applicable, describe the population and methods used to elicit preferences for outcomes. | Method section ‘Outcomes’. |
| Estimating resources and costs | 13a | *Single study-based economic evaluation:* Describe approaches used to estimate resource use associated with the alternative interventions. Describe primary or secondary research methods for valuing each resource item in terms of its unit cost. Describe any adjustments made to approximate to opportunity costs. | Not Applicable. |
|  | 13b | *Model-based economic evaluation:* Describe approaches and data sources used to estimate resource use associated with model health states. Describe primary or secondary research methods for valuing each resource item in terms of its unit cost. Describe any adjustments made to approximate to opportunity costs. | Supplement section ‘Supplement Text 1’. |
| Currency, price date, and conversion | 14 | Report the dates of the estimated resource quantities and unit costs. Describe methods for adjusting estimated unit costs to the year of reported costs if necessary. Describe methods for converting costs into a common currency base and the exchange rate. | Method section ‘Utility data’. |
| Choice of model | 15 | Describe and give reasons for the specific type of decision-analytical model used. Providing a figure to show model structure is strongly recommended. | Method section ‘Microsimulation state-transition model’; Figure 1. |
| Assumptions | 16 | Describe all structural or other assumptions underpinning the decision-analytical model. | Method section ‘Microsimulation state-transition model’. |
| Analytical methods | 17 | Describe all analytical methods supporting the evaluation. This could include methods for dealing with skewed, missing, or censored data; extrapolation methods; methods for pooling data; approaches to validate or make adjustments (such as half cycle corrections) to a model; and methods for handling population heterogeneity and uncertainty. | Method section ‘Sensitivity analysis’. |
| **Results** | | | |
| Study parameters | 18 | Report the values, ranges, references, and, if used, probability distributions for all parameters. Report reasons or sources for distributions used to represent uncertainty where appropriate. Providing a table to show the input values is strongly recommended. | Method; Table 1. |
| Incremental costs and outcomes | 19 | For each intervention, report mean values for the main categories of estimated costs and outcomes of interest, as well as mean differences between the comparator groups. If applicable, report incremental cost-effectiveness ratios. | Method; Table 3. |
| Characterising uncertainty | 20a | *Single study-based economic evaluation:* Describe the effects of sampling uncertainty for the estimated incremental cost and incremental effectiveness parameters, together with the impact of methodological assumptions (such as discount rate, study perspective). | Not applicable. |
|  | 20b | *Model-based economic evaluation:* Describe the effects on the results of uncertainty for all input parameters, and uncertainty related to the structure of the model and assumptions. | Method section ‘Sensitivity analysis’. |
| Characterising heterogeneity | 21 | If applicable, report differences in costs, outcomes, or cost-effectiveness that can be explained by variations between subgroups of patients with different baseline characteristics or other observed variability in effects that are not reducible by more information. | Not applicable. |
| **Discussion** | | | |
| Study findings, limitations, generalisability, and current knowledge | 22 | Summarise key study findings and describe how they support the conclusions reached. Discuss limitations and the generalisability of the findings and how the findings fit with current knowledge. | Discussion. |
| **Other** | | | |
| Source of funding | 23 | Describe how the study was funded and the role of the funder in the identification, design, conduct, and reporting of the analysis. Describe other non-monetary sources of support. | Conflict of Interest Disclosures. |
| Conflicts of interest | 24 | Describe any potential for conflict of interest of study contributors in accordance with journal policy. In the absence of a journal policy, we recommend authors comply with International Committee of Medical Journal Editors recommendations. | Source of funding. |

**Supplement Table 2** Comparison of weight distribution of CHIS and simulated cohort in 2015.*

|  | CHIS cohort in 2015 | Simulated cohort in 2015 |
| --- | --- | --- |
| Population size | 20511 | 20000 |
| Normal weight, n (%) | 7613(37.1%) | 6374(32.5%) |
| Overweight, n (%) | 7104(34.6%) | 6409(31.6%) |
| Obese, n (%) | 5794(28.2%) | 7217(35.9%) |

*Estimates were weighted for CHIS. Mean (SD) for continuous variables, N (%) for categorical variables.

**Supplement Table 3** Comparison of weight distribution and clinical outcomes of CHIS and the simulated cohort in 2019, % and 95%CI.*

|  | California population in 2019 | Simulated cohort in 2019 |
| --- | --- | --- |
| Normal weight | 36.9% | 39.1% (38.9%-39.4%) |
| Overweight | 34.2% | 34.9% (34.8%-34.9%) |
| Obese | 26.2% | 26.0% (25.8%-26.3%) |
| Diabetes | 10% | 9% (8.3%-10.0%) |
| MI | 3.1% | 4.7% (4.3%-5.1%) |
| Stroke | 2.8% | 3.1% (2.8%-3.2%) |
| ESRD | 0.4% | 0.1% (0.1%-0.2%) |
| Death | 0.7% | 1.1% (1.0%-1.1%) |

*Estimates were weighted for CHIS.

**Supplement Table 4** Comparison of weight distribution and clinical outcomes of NHANES and the simulated NHANES cohort in 2019, % and 95%CI.

|  | NHANES population in 2019 | Simulated cohort in 2019 |
| --- | --- | --- |
| Normal weight | 30.0% | 29.1% (27.9%-30.4%) |
| Overweight | 31.1% | 20.9% (17.9%-23.9%) |
| Obese | 42.5% | 49.9% (49.9%-50.0%) |
| Diabetes | 10.5% | 8.5% (7.7%-9.3%) |
| MI | 3.0% | 3.5% (3.1%-3.9%) |
| Stroke | 3.4% | 2.8% (2.3%-3.3%) |
| ESRD | 0.1% | 0.1% (0.1%-0.2%) |
| Death | 0.7% | 1.0% (0.9%-1.0%) |

**Supplement Table 5** Summary of comparativeness under status quo and soda tax for a hypothetical cohort of 20,000 Californian adults in 20 years.*

|  | **1-cent-per ounce soda tax** | **Status quo** | **Number of cases prevented** |
| --- | --- | --- | --- |
| **Number of overweight cases** | 7200(6362, 9625) | 9445(8834, 9811) | 2245(-42, 4532) |
| **Number of obesity cases** | 7254(7200, 7645) | 7591(7325, 8011) | 337(-130, 804) |
| **Number of diabetes cases** | 866(710, 1056) | 1123(790, 1660) | 257(-227, 741) |
| **Number of stroke cases** | 306(246, 370) | 437(285, 696) | 131(-94, 356) |
| **Number of MI cases** | 567(470, 676) | 675(515, 897) | 108(-113, 329) |
| **Number of ESRD cases** | 204(175, 235) | 198(169, 229) | -6(-49, 37) |
| **Number of death cases** | 1385(1251, 1526) | 1871(1344, 2734) | 486(-278, 1250) |

* Mean and 95%CI. MI, myocardial infarction, ESRD, end stage renal disease.

**Supplement Table 6** Summary of cost effectiveness under status quo and soda tax for a hypothetical cohort of 20,000 Californian adults in 20 years.*

|  | **Status quo** | **1-cent-per ounce soda tax** |
| --- | --- | --- |
| **Total healthcare costs** | $463,015,255 (412,704,052 to 512,755,695) | $362,226,767 (287,399,913 to 465,815,126) |
| **Healthcare cost savings per person** | - | $5,039 |
| **Total costs, government perspective** | $463,015,255 (412,704,052 to 512,755,695) | -$12,725,763,529 (-12,800,590,384 to -12,622,175,170) |
| **Total QALYs** | 265,917(264,819 to 266,999) | 267,895(265,325 to 272,023) |
| **Incremental QALYs** | - | 1,979 |
| **Incremental cost, healthcare perspective** | - | -$100,788,488 |
| **Incremental cost, government perspective** | - | -$13,188,778,784 |
| **ICER, healthcare perspective** | - | -124,839 (-1,151,983 to 557,660) |
| **ICER, government perspective** | - | -14,668,299 (-112,205,426 to -5,7206,440) |

Mean and 95%CI. QALY, quality adjusted life years; ICER, incremental cost-effectiveness ratio.

Negative costs represented that there were net revenues.

Costs were in 2022 dollar.

**References**

1. Sundbøll J, Horváth-Puhó E, Schmidt M, et al. Long-Term Risk of Stroke in Myocardial Infarction Survivors. *Stroke*. 2016;47(7):1727-1733. doi:10.1161/STROKEAHA.116.013321

2. Engstad T, Viitanen M, Arnesen E. Predictors of Death Among Long-Term Stroke Survivors. *Stroke*. 2003;34(12):2876-2880. doi:10.1161/01.STR.0000101751.20118.C1

3. Cawley J, Frisvold D, Hill A, Jones D. Oakland’s sugar-sweetened beverage tax: Impacts on prices, purchases and consumption by adults and children. *Economics & Human Biology*. 2020;37:100865. doi:10.1016/j.ehb.2020.100865

4. Hall KD, Sacks G, Chandramohan D, et al. Quantification of the effect of energy imbalance on bodyweight. *The Lancet*. 2011;378(9793):826-837. doi:10.1016/S0140-6736(11)60812-X

5. Gidwani R, Russell LB. Estimating Transition Probabilities from Published Evidence: A Tutorial for Decision Modelers. *Pharmacoeconomics*. Published online August 14, 2020:1-12. doi:10.1007/s40273-020-00937-z

6. VanderWeele TJ. Optimal approximate conversions of odds ratios and hazard ratios to risk ratios. *Biometrics*. 2020;76(3):746-752. doi:10.1111/biom.13197

7. VanderWeele T. On a square-root transformation of the odds ratio for a common outcome. *Epidemiology*. 2017;28(6):e58-e60. doi:10.1097/EDE.0000000000000733
